# Impeding and facilitating factors for the implementation of alcohol interventions in hospitals: a qualitative and exploratory study among Dutch healthcare professionals

**DOI:** 10.1186/s12913-021-07412-1

**Published:** 2022-01-02

**Authors:** Nathalie Kools, Ien van de Goor, Rob H. L. M. Bovens, Dike van de Mheen, Andrea D. Rozema

**Affiliations:** Tranzo Scientific Center for Care and Wellbeing, Tilburg School of Social and Behavioral Sciences, Tilburg University, PO box 90153, 5000 LE Tilburg, The Netherlands

**Keywords:** Alcohol drinking, Alcoholism, Attitude of health personnel, Early medical intervention, General hospitals

## Abstract

**Background:**

Non-moderated alcohol use is more prevalent among hospitalized patients compared to the general population. However, many hospitals fail to find and intervene with people with alcohol problems. We aimed to conduct an exploration of impeding and facilitating factors experienced by healthcare professionals in implementation of alcohol interventions in Dutch general hospitals. In addition, we explored the alcohol interventions used in the selected hospitals and involved stakeholders.

**Methods:**

Through a qualitative study, semi-structured telephone interviews were conducted with twenty healthcare professionals working in or in collaboration with six different general hospitals.

**Results:**

Healthcare professionals indicated impeding and facilitating factors in the areas of motivation, knowledge and skills, patient characteristics, protocol, internal and external collaboration/support, resources, role suitability and societal support. Five different categories of approaches to identify and intervene with non-moderated alcohol use and 18 involved stakeholders from both inside and outside the hospital were found.

**Conclusions:**

Implementation of alcohol interventions for patients in Dutch general hospitals still seems to be in its infancy. Respondents emphasized the importance of one clear protocol on how to tackle alcohol problems within their hospital, repeated training on alcohol-related knowledge and skills, (clinical) “champions” that support healthcare professionals and developing and maintaining collaborations with stakeholders within and outside the hospital.

## Background

Reducing harm due to alcohol use seems to remain a challenge in many countries, as the prevailing and accepted social norm continues to be drinking rather than not drinking [1]. What makes reducing harm due to alcohol use even more difficult is that there exists no international uniformity in terms of drinking guidelines and what is considered a standard drink [2, 3]. That is, what is considered hazardous drinking in one country may be considered acceptable in another country. In the Netherlands, 8.2% of the adult population is an excessive drinker (drinks more than twenty-one or fourteen glasses in total per week, respectively for men and women) and 9.0% is a heavy drinker (drinks more than six or four glasses during one occasion, respectively for men and women) [4].

Non-moderated alcohol use is more common among hospital patients^1^ than in the general population. For example, research shows that 16 to 26% of hospital patients from different hospital departments are found to have risky or harmful alcohol use when screened for it [5–8]. Hospitals therefore seem to be an appropriate place to detect and intervene with people with non-moderated alcohol use.

Within the health care sector, various approaches have been developed to identify and intervene with non-moderated alcohol use. For example, “screening, brief intervention and referral to treatment” (SBIRT) is a well-known approach to identifying non-moderated alcohol use early and providing appropriate levels of treatment [9]. However, such approaches are not (yet) always implemented within hospitals [10], which means that many hospital professionals fail to identify and intervene with people with alcohol problems.

To find out why such alcohol interventions^2^ are not yet always implemented in hospitals and to inform future implementations, impeding and facilitating factors need to be known of this specific context. For example, previous international research within hospitals found that lack of knowledge, inadequate time and resources and personal discomfort in confronting patients with their alcohol use were important impeding factors for implementing alcohol interventions within hospital settings [11]. Important facilitating factors included improved electronic health record features (e.g., templates for screening, electronic reminders and assessment-related consultation orders) and improved knowledge and skills of and collaboration between health care professionals [12, 13].

This is the first study to map the Dutch situation regarding alcohol interventions in hospitals. As the Dutch context differs in healthcare organization, structure and funding from those abroad [14], it is interesting to investigate what type of impeding and facilitating factors are found here. The aim of this study was to conduct an initial exploration of impeding and facilitating factors experienced by healthcare professionals in the implementation of alcohol interventions in Dutch hospitals. As little is known about the Dutch situation, we also inventoried which alcohol interventions are used by hospitals in the selected sample and which stakeholders are involved. Finally, a practical interest within the present study was to inform a Dutch action plan for improving the implementation of alcohol interventions in Dutch hospitals.

## Methods

### Study setting

We conducted qualitative, semi-structured interviews with twenty healthcare professionals working in, or in collaboration with, various general hospitals throughout the Netherlands. In total, six different general hospitals (of a total of 69 in the Netherlands) and six different organizations (i.e., social work, psychiatry and addiction care organizations), working in collaboration with hospitals were included.

### Participants

We used a purposive sampling method [15] for the recruitment of respondents, consulting the national working group “Secondary Care” (Tweedelijn) of the Dutch Partnership Early Detection of Alcohol (Samenwerkingsverband Vroegsignalering Alcoholproblematiek (SVA)). This working group consists of representatives from various organizations that are involved in alcohol interventions in secondary care, including the prevention department of Verslavingskunde Nederland (national addiction expertise network). These representatives had a good overview of available hospital interventions in the Netherlands in the field of alcohol problems and therefore served as a source of information about which hospitals and health care professionals could be included in order to get a first impression of the Dutch situation. Some hospitals were contacted and asked to provide professionals that were active in alcohol interventions. Other professionals were directly contacted as they were seen as experts in the intervention field within their hospital or organization. This resulted in a heterogeneous group of health care professionals working in different departments and disciplines. The sample selection showed a good geographical spread across the Netherlands and the sample size was deemed suitable for our purpose of conducting a first exploration (as opposed to a survey at all hospitals) of perceived impeding and facilitating factors, and of existing alcohol interventions and involved stakeholders.

We contacted all selected professionals by email, informed them about the study and research objectives, and invited them for an interview. In total, we contacted 27 professionals, of whom 20 participated in the study. For the seven who did not participate, lack of time was the main reason for non-participation. Among the twenty respondents interviewed, ten were female (50.0%) and the average age was 45.3 years (*SD* = 12.0). All of the respondents were White (100.0%). Other participant characteristics are shown in Table 1.

**Table 1:** Participant characteristics

No.	Role	Sex	Department	Organization type
1^a^	Nurse	F	Gastroenterology	Top clinical hospital 1
2^a^	Attending physician	M	Gastroenterology	Top clinical hospital 1
3^a^	Social worker	M	–	Social work organization
4^b^	Psychiatric nurse	F	–	Psychiatry organization 1
5^b^	Psychiatric nurse practitioner	M	–	Psychiatry organization 2
6^b^	Psychiatric nurse	M	–	Addiction care organization 1
7^b^	Prevention officer	M	–	Addiction care organization 2
8^b^	Attending physician	F	Emergency	Top clinical hospital 2
9^b^	Resident physician	M	Emergency	Top clinical hospital 2
10^b^	Attending physician	F	Emergency	Academic hospital 1
11^b^	Nurse practitioner	M	Emergency	Academic hospital 1
12^b^	Attending physician	F	Emergency	Academic hospital 1
13^b^	Attending physician	F	Emergency	Top clinical hospital 3
14^b^	Psychiatric nurse	M	–	Psychiatry organization 3
15^b^	Psychiatric nurse	F	–	Psychiatry organization 3
16^b^	Attending physician	M	Otorhinolaryngology	Academic hospital 2
17^b^	Health care manager/Nurse	M	Emergency	Top clinical hospital 3
18^b^	Attending physician	F	Otorhinolaryngology	Community hospital
19^b^	Nurse	F	Emergency	Academic hospital 1
20^b^	Resident physician	F	Internal medicine	Top clinical hospital 2

^a^Face to face interview, ^b^Telephone interview; ^c^Column ‘Department’ only applies to respondents working in a hospital, respondents working in other organizations do not have a department mentioned (marked with a hyphen)

*F* female, *M* male

### Procedures

Ethical approval was granted by the Ethics Review Board of Tilburg University (EC-2019.94). Prior to the interviews, all twenty respondents provided informed consent. All interviews were conducted by the first author (NK) a female junior researcher (MSc) with some previous interview experience. Data collection involved in-depth, semi-structured interviews, using an interview guide. Interviews included three main sections: (1) existing alcohol interventions in the respondent’s hospital, (2) stakeholders involved in this, and (3) factors that impede or facilitate implementation. Examples of interview questions are: *“What barriers have you encountered in the implementation of alcohol interventions?”* and *“To what extent do professionals have the knowledge and skills to discuss alcohol problems?”* Most interviews were conducted by telephone (75.0%), and the others face-to-face. Data retrieved from telephone interviews appear to have similar quality to face-to-face interviews [16]. The face-to-face interviews were conducted at one hospital in a private room. No other persons were present at any interview beyond the individual respondents and interviewer. All interviews were conducted between November 2019 and January 2020. Interviews lasted an average of 50 min (*SD* = 14; *range* 29–71) and were conducted in Dutch. All interviews were audio-recorded.

### Analysis

Interviews were transcribed verbatim by professional transcriptionists. Transcripts were qualitatively analyzed using reflexive thematic analysis. Following an inductive and semantic approach, codings and theme developments were driven by the data and reflected the explicit content of the data [16, 17]. Transcripts were coded by the first author (NK) in the software package ATLAS-Ti 8 [18], distinguishing between the three research questions by the classifications ‘alcohol intervention ‘, ‘stakeholder’ or ‘factors that may impede or facilitate implementation’. Thirty percent of the transcripts were coded independently by the last author (ADR) and then compared and discussed until agreement was reached. Code groups were then created, which were classified into 24 general themes. To clarify in which area themes were identified, the general themes were classified into four categories (individual level, protocol level, organization level, and society level), based in part on the measurement instrument for determinants of innovation (MIDI) [19]. Finally, in consultation with all co-authors, the appropriateness of the developed themes and their classification were discussed and adjusted as necessary.

## Results

The results are divided into three sections: factors that may impede or facilitate the implementation of alcohol interventions, identified alcohol interventions, and stakeholders involved.

### Factors that may impede or facilitate implementation of alcohol interventions

In total, eight factors were identified: motivation, knowledge and skills, patient characteristics, protocol, collaboration/support, resources, role responsibility, and societal support. These factors generally consisted of two sides of the same coin: the impeding side (if they are absent) and facilitating side (if they are present) (Table 2).

**Table 2:** Impeding or facilitating factors for implementation

	Impeding factors		Facilitating factors	
Level	Factor	Code	Factor	Code
**Protocol level**	1. Unclear protocol	1.1. Lack of protocol 1.2. Too many protocols 1.3. Time-consuming intervention actions after screening 1.4. Difficulties due to patient privacy	1. Well-developed protocol	1.1. Presence of protocol 1.2. User-friendly protocol 1.3. Embedded in the system 1.4. Incorporating lifestyle-wide intervention approaches
**Individual level**	1. Lack of professionals’ motivation	1.1. Decrease in attention to theme 1.2. Forgotten 1.3. Distrust in usefulness and effectiveness 1.4. Perception that it is not their job 1.5. Irrelevant for care demand of patient 1.6. Alcohol not recognized as problem 1.7. Resistance care professionals 1.8. Resistance (partnerships) physicians 1.9. Resistance pharmacists 1.10. Resistance general practitioners	1. Enhancing professionals’ motivation	1.1. Emphasizing interventions’ importance 1.2. Feedback on effect of intervention 1.3. Presence of clinical “champion” 1.4. Peer coaching 1.5. Reminding each other 1.6. Showing perseverance 1.7. Enhancing feelings of responsibility 1.8. Involving professionals in protocol development 1.9. Higher motivation present in specific departments 1.10. Higher motivation present if relevant to patient care demand 1.11. Commitment project group
	2. Lack of professionals’ knowledge and skills	2.1. Lack of knowledge regarding alcohol (problems) 2.2. Not knowing how to start conversation 2.3. Loss of knowledge due to staff turnover 2.4. Not knowing where to refer to	2. Enhance professionals’ knowledge and skills	2.1. Training 2.2. Knowing how to start conversation 2.3. Gaining experience with alcohol problem patients 2.4. Limited staff turnover
	3. Difficulties in patient contact	3.1. Patient unaware of the problem 3.2. Patient still intoxicated 3.3. Patient does not answer honestly 3.4. Language/cultural barriers 3.5. Patient aggressive or insulting 3.6. Patient unmotivated 3.7. Multiple problems patient	3. Support from patient	3.1. Patient receptive to alcohol intervention 3.2. Involvement patients’ social network
**Organizational level**	1. Poor collaboration/support	1.1. Difficulties involving general practitioner 1.2. Hard to keep multidisciplinary project group together 1.3. Absence of physician during consultations 1.4. Vulnerable transfer of (patient) information after discharge 1.5. Dependence on operating hours and schedule 1.6. Resistance hospital management	1. Good collaboration/support	1.1. Knowing the network 1.2. Cooperating in multidisciplinary project group 1.3. Presence of physician during consultations 1.4. Having collaboration agreements between parties 1.5. Collaboration between hospital departments 1.6. Collaboration in the health care chain (parties outside hospital) 1.7. Collaboration general practitioner 1.8. Commitment hospital management
	2. Lack of resources	2.1. Lack of finances 2.2. Lack of staff 2.3. Lack of time 2.4. Limited care possibilities due to insurers’ Diagnosis-Treatment-Combinations (DTC)	2. Sufficient resources	2.1. Sufficient finances 2.2. Sufficient staff 2.3. Sufficient time 2.4. Hiring counsellor/task-specific employee 2.5. Insure patients holistically, without Diagnosis-Treatment-Combinations (DTC)
	3. Unclear role responsibility	3.1. Disagreements about role responsibilities	3. Compatible with role responsibility	3.1. Compatible with role of physician 3.2. Compatible with role of nurse 3.3. Compatible with role of general practitioner
**Societal level**	1. Lack of societal support	1.1. Lack of national information 1.2. Negative image of addiction care 1.3. Social acceptance of drinking alcohol 1.4. Intervention perceived as premature	1. Societal support	1.1. National information 1.2. Governmental regulations

#### Protocol level

At protocol level, respondents mentioned the importance of having one clear protocol with user-friendly action perspectives. Respondents mentioned that the distribution of responsibilities regarding the implementation of alcohol interventions should be clearly defined and recorded in protocols. In addition, protocols should state with whom in the (health care) chain can be collaborated. As a solution for professionals’ lack of time, the use of self-report questionnaires to screen patients and a well-developed embedded electronic system were suggested. Finally, in addition to making a protocol mandatory, it was mentioned that end-users (healthcare professionals) should be involved in its drafting to avoid resistance in implementation

*'I think the main problem is just that we don't do it, that there is not something, a program, project or a protocol or something that goes with it. And then you have to go out and invent it yourself when this happens.'* [R16; Otorhinolaryngology physician]

*‘So the moment you have a suspicion, whether or not based on such a tool, and you can refer that to someone in a very easy logistical way, I think that has the highest chance of success. So the tool should not be too complicated, it should not be time consuming and the logistics should be good.'* [R20; Internal medicine physician assistant]

#### Individual level

At the individual level, respondents mentioned that professionals themselves play a crucial role in the successful implementation of alcohol interventions, more specifically their motivation, knowledge, and skills. Respondents mentioned that motivation is sometimes already present in specific contexts, for example in departments where alcohol problems are relatively common (e.g. gastrointestinal and liver diseases). Similarly, professionals seem (more) motivated regarding patients whose clinical picture is clearly related to alcohol. In other contexts, efforts seem necessary to convince professionals of their responsibility and the importance of alcohol interventions.

*‘If you want to get people to do something, you have to show its usefulness, especially if you ask people to do this in a very busy job. Then they just have to fully see that what they have to do in addition is valuable, and if they don't see that, then they just won't do it.’* R8; Emergency physician]

As for professionals’ knowledge and skills, respondents mentioned that it is important not only to have procedural knowledge about the interventions, but also to have knowledge about the risks and injuries related to alcohol use, motivational interviewing techniques and ways of dealing with taboos in conversations with patients. Respondents mentioned that (repeated) training is crucial to ensure that professionals have this knowledge and skills and maintain it, regardless of staff turnover and reorganizations.

*‘I think that a lot of people don't even know what kind of, uhm, how the problems can sometimes come about. What the problem entails exactly, what an addiction is in the first place and why it is that people sometimes do not get rid of their addiction.’* [R14; Psychiatry organization; Psychiatric nurse]

*'Nurses find it very difficult to start that conversation, feel burdened, fear that they will open up a can of worms and that they don't know how to respond.'* [R1; Gastroenterology nurse]

Respondents also mentioned that professionals may simply forget to conduct alcohol interventions in the busyness of their daily work. Therefore, the presence of (clinical) “champions,” peer coaching, and reminders devices were mentioned as being helpful. In addition, respondents mentioned that the “champion” should ideally be from one’s own department, and be someone who is known, present on a daily basis and has some authority in terms of years of experience. Furthermore, participants mentioned that the support of various parties is necessary.

*‘We had a nurse practitioner at the time and that was more or less the driving force behind the entire process. He was on top of it, and he also made printouts every week of what was asked. At one point, he started calling people who had an intervention, whether they had done something with their alcohol use. You see, if you have someone, a booster who follows all that, and also publishes at some point, yes, it’s a … a kind of motivational story in itself’* [R17; Emergency health care manager/nurse]

Finally, respondents mentioned some alcohol-related patient characteristics that complicate implementation, such as lying about alcohol use or being too drunk to have a conversation. Patients’ receptivity to alcohol screening and having a dialogue were mentioned as helpful. For example, respondents noted that some patients perceive alcohol screening and questions regarding alcohol as a normal part of the overall “hospitalization package.”

#### Organizational level

At the organizational level, facilitating implementation with adequate resources seems crucial. Respondents mentioned having sufficient time for alcohol interventions as most crucial and described that screenings, opening a dialogue and the referral process are often too time consuming in the short duration of hospitalizations. Respondents suggested hiring or appointing special-task employees who could take on intervention tasks. This specific set of tasks, according to respondents, could be integrated into their work for virtually all disciplines, including nurses, nurse specialists, addiction specialists, (medical) social workers, consultative psychiatric nurses and experts by experience.

*‘But you could very well have someone working here 24/7 who can do work on multiple fronts, including addiction screening, and then we are also talking about tobacco and alcohol and drugs, of course, and talk to those people directly, but also call them back, see if they've thought about it, see if a referral makes sense, talk to the GP...*' [R8; Emergency physician]

In addition, respondents mentioned that it should be clear with whom in the (health care) chain can be collaborated and that this could be achieved through collaboration agreements and by simply getting to know each other, for example through network meetings. Respondents named, for example, social work, addiction care and ambulance services as relevant cooperation partners. On the other hand, poor collaboration was mentioned as an important impeding factor, for example due to long waiting lists for referral, the absence of collaborating partners at peak times (e.g. weekends or nights) or the absence of physicians during multidisciplinary meetings due to a busy schedule.

*'We also got to know each other. If you know each other, you also know how the lines run, where you can refer people to'* [R17; Emergency health care manager/nurse]

#### Societal level

At the societal level, respondents mentioned that supporting government regulations could facilitate implementation, for example, by regulations that make alcohol interventions in hospitals mandatory and by a stricter approach with regard to alcohol use in general (e.g., making alcohol more expensive). In addition, it was mentioned that the general population should be educated more about the risks and related harms of alcohol use. Respondents also mentioned that current social norms need to be broken, so that the new norm becomes not to drink alcohol, rather than to drink it. Finally, according to respondents, efforts should be made to change the negative image of addiction care.

*‘Or it should become a performance indicator. So if it becomes a legal requirement, then they should.'* [R5, Addiction care organization; Psychiatric nurse specialist]

*'And I also think um, with some people it's anyway, social acceptance also ensures that you don't acknowledge it as a problem, and also don't recognize it as a problem.'* [R14; Psychiatry organization; Psychiatric nurse]

### Identified alcohol interventions

Based on respondents’ answers, the identified alcohol interventions were divided into five categories (Table 3). The first category of interventions was the use of information materials. In several hospitals, information leaflets were given to patients (whether or not after an alcohol screening), which contained a variety of information about (risks of) alcohol use and ways to seek help. In addition, posters were used in waiting rooms. These included posters that informed patients that their alcohol use could be asked about and posters that informed patients about the national campaign “IkPas”, which is a Dutch alcohol awareness campaign where people put their alcohol use on hold for 30 or 40 days.

**Table 3:** Identified alcohol interventions

Category	Alcohol intervention
1. Information materials	1.1. Information leaflet 1.2. Poster
2. Screening	2.1. Screening intuitively when suspecting non-moderated alcohol use 2.2. Generally asking for alcohol use during medical history or triage 2.3. With screening tool in Electronic Health Record 2.4. With blood test
3. Opening a dialogue with patients	3.1. Lifestyle education conversation 3.2. Video-intervention 3.3. Advice about contacting a general practitioner 3.4. Advice about contacting addiction care
4. Consultations between professionals in the hospital	4.1. Multidisciplinary consultation 4.2. Quality review during clinical handover
5. Involvement of (external) parties	5.1. Involving the general practitioner in consultation with patient 5.2. Involving psychiatry in consultation with patient 5.3. Referring to addiction care in consultation with patient

The second category of interventions was to conduct alcohol screenings. Approaches of the screening varied widely, from intuitive actions to systematic screening. One approach was to ask about alcohol use during history or triage. This was done intuitively when health professionals suspected non-moderated alcohol use (e.g., when patients looked like they drank a lot of alcohol or when patients were frequently readmitted), or in a standardized manner using a screening tool (e.g., AUDIT(−C)) that was included in the electronic health record. Another approach was screening based on blood tests.

The third category of interventions was opening a dialogue with patients. One approach was to provide general lifestyle education during these conversations, sometimes using motivational interviewing techniques. Motivational interviewing is a “client-centered, directive therapeutic style to enhance readiness for change by helping clients explore and resolve ambivalence” [20]. Another approach was to provide digital health education by showing a video on a tablet, which included motivational interviewing techniques. A final approach was to provide patients with substantiated advice to contact their primary care physician or to seek addiction care.

The fourth category of interventions was consultations between professionals in the hospital. One approach was to organize multidisciplinary consultations, involving different disciplines to discuss specific patients with alcohol problems. In one hospital, a social worker from outside the hospital was routinely present at these consultations in addition to professionals from the hospital. Another approach for consultations was to address the alcohol topic in the quality reviews during clinical transfers. This was done by verbally checking whether certain follow-up steps around alcohol issues had been considered, such as contacting addiction services.

The fifth and final category of interventions was to involve external parties from outside the hospital, provided that patients consented. This was done by mentioning patients’ alcohol use in discharge letters to patients’ general practitioners. GPs were also contacted by telephone to request information about the patient or to transfer further treatment of the non-moderated alcohol use. In addition, psychiatry was involved through manual actions or through automatic messages as soon as patients scored positive on alcohol screenings. Furthermore, if patients consented, patients were referred to addiction services through referral letters. Patients were then contacted by the addiction clinic or had to contact them themselves for their appointment. A final approach was to have standardized referral procedures for patients whose intoxication was the reason for hospital visit.

### Identified involved stakeholders

In total, 18 involved stakeholders were identified, distinguishing between stakeholders inside (8) and outside (10) hospitals. An overview is shown in Table 4. Stakeholders within the hospital included healthcare professionals (e.g., nurses, physicians, and medical social workers), hospital management, and physician partnerships. Stakeholders outside the hospital could be subdivided into healthcare professionals outside the hospital (e.g., psychiatry organizations, addiction care organizations, general practitioners, and ambulance services), facilitating stakeholders (e.g., social workers, home care services, and health insurers), and people in the private sphere of patients (e.g., family and friends). Subcategories of stakeholders could be identified within psychiatry organizations, including crisis services and outreach teams.

**Table 4:** Identified involved stakeholders

Stakeholder level	Stakeholders
1. Within hospital	1.1. Physicians (and physician partnerships)
	1.2. Resident physicians
	1.3. Nurses
	1.4. Nurse specialists
	1.5. Psychiatric Consultation Service/Psychiatric Department
	1.6. Dieticians
	1.7. Medical social workers
	1.8. Management of hospital
2. Outside hospital	2.1. Ambulance services
	2.2. Addiction care organizations
	2.3. General practitioners
	2.4. Psychiatry/general mental health care workers
	2.5. Social workers
	2.6. Home care services
	2.7. Municipal health services
	2.8. Health insurers
	2.9. Safe at home organisztions^a^
	2.10. Patient relatives

^a^Safe at home organizations (Dutch: Veilig Thuis) offer advice and support regarding domestic violence and child abuse

## Discussion

The purpose of this study was to gain insight into impeding and facilitating factors experienced by healthcare professionals in the implementation of alcohol interventions in Dutch hospitals, as well as which alcohol interventions these healthcare professionals use and which stakeholders are involved.

Respondents indicated several important factors for implementation, including having one user-friendly protocol in their hospital, training and a clinical “champion.” Respondents also cited time and resources, close collaborations between parties in the (health care) chain and government regulations as important factors. Five different types of alcohol interventions were identified: information materials, screening, opening a dialogue with patients, consultations between professionals in the hospital and involving (external) parties. Parties involved in these interventions included both internal stakeholders (e.g., hospital staff, psychiatry caregivers and hospital management) and external stakeholders (e.g., family physician, addiction counsellors and social workers).

Many factors in the current study are consistent with previous international research within hospitals: improved knowledge and skills, increased motivation and sense of responsibility (e.g., through reminders), patient receptivity, and development of a single clear and user-friendly protocol [12, 13, 21–23]. Strikingly, having an environment where physician and patient have sufficient privacy to discuss alcohol use is frequently mentioned in the international literature [11, 13, 22, 23], but this factor was not specifically mentioned in this study. However, we did find that healthcare professionals found it difficult to initiate the conversation because of the taboo surrounding alcohol problems. Possibly this aspect of insufficient privacy is part of it.

In addition, in previous international research, the factor “lack of support” frequently comes up, but then this factor only includes (the lack of) support from colleagues within hospital departments [11]. In this study, the lack of support from hospital management was added as an important impeding factor. Moreover, this study also mentioned the importance of collaboration with parties outside the hospital, with parties such as general practitioners, addiction treatment facilities, and social workers. Particularly the collaboration with the general practitioner is new/different from the international literature here [11]. This is probably because the Dutch healthcare system is organized in such a way that general practitioners act as gate-keepers for the hospital, in contrast to many other countries [14]..

In addition, it is notable that previous international research did not mention factors at the societal level [11], such as (the lack of) national education about alcohol risks to the general population, social norms and the negative image of addiction care, which were mentioned in the current study. These factors may partly influence the extent to which healthcare professionals implement alcohol interventions and the degree to which patients are receptive to them. More research is needed on the extent to which social norms and attitudes of both healthcare professionals and patients influence the implementation of alcohol interventions in a hospital setting.

The various alcohol interventions as found in the current study appear to have many similarities to SBIRT practices [9]. At the European level, the first two categories of SBIRT (i.e., alcohol screening and brief intervention (SBI)) seem to be implemented in general hospitals to some degree in the United Kingdom (UK), Spain and Switzerland [24–26]. Interventions that we did not find in the Netherlands but that are implemented in UK hospitals are multidisciplinary Alcohol Care Teams (ACTs), which offer integrated alcohol treatment across primary, secondary and community care [27]. ACTs mostly resemble the multidisciplinary consultation intervention found in the present study, but these seem to be less widely deployed than ACTs. Nevertheless, although at the international level alcohol intervention programs often do exist, the literature shows that most of them are underutilized, rarely implemented, or limited to primary health care [24]. Our study confirms this finding, as we find that the thoroughness with which alcohol interventions are implemented for patients in Dutch hospitals varied widely.

Finally, in this study we found that many different stakeholders are involved in the implementation of alcohol interventions in hospitals. However, involvement alone does not directly imply interprofessional collaboration, whereas this is considered essential for improving health and patient care [28]. It is therefore important to strengthen interprofessional collaboration through, for example, shared goals and visions, multidisciplinary meetings, and interprofessional education [28, 29], factors that were also mentioned as promoting in this study.

A limitation of this study is the recruitment through a purposive sampling method drawn from the network of the SVA working group “Secondary Care”. Although the representatives of the prevention departments within Verslavingskunde Nederland in particular have a good overview of the available hospital interventions in the field of alcohol in the Netherlands, this sample is not fully representative. However, the inclusion of different hospital departments throughout the Netherlands provided a heterogeneous group of healthcare professionals, resulting in a rich and detailed dataset.

## Conclusions

Implementation of alcohol interventions for patients with alcohol problems in Dutch hospitals still seems to be in its infancy. As the Dutch context differs in healthcare organization, structure and funding from those abroad [14], it was important to investigate what type of impeding and facilitating factors are found here in order to inform future implementations. Identified important impeding and facilitating factors for implementing alcohol interventions in Dutch hospitals largely correspond to those found in international literature. An important point of attention for the Dutch situation is to develop and maintain collaborations with stakeholders both inside and outside the hospital. More research needs to be conducted on the effectiveness of the found facilitating factors in (Dutch) hospitals. Finally, the findings of this study can provide further information regarding alcohol intervention strategies at the international level, which in turn might result in a better care chain for patients with alcohol problems and in further reduction of non-moderated alcohol use.

## Data Availability

The datasets generated and analyzed during are available from the corresponding author on reasonable request.

## Abbreviations

NK: Nathalie Kools (study author)
ADR: Andrea D. Rozema (study author)

## Footnotes

1 By hospitals we mean general health care hospitals (both inpatient and emergency departments), so no psychiatric hospitals.

2 By alcohol interventions we mean all types of approaches to alcohol problems, ranging from screenings to referral of patients.
